# Multivariate performance indices in federal university hospitals: an exploratory analysis using principal component analysis

**DOI:** 10.1108/IJHCQA-10-2025-0156

**Published:** 2026-03-11

**Authors:** Maria Gabriela Mendonça Peixoto, Gustavo Alves de Melo, Maria Cristina Angélico Mendonça, Marcel Andreotti Musetti, André Luiz Marques Serrano, Denisie Ellen de Iovanna, Matheus de Sousa Pereira, Thiago Henrique Nogueira, Francisco Gabriel Gomes Dias

**Affiliations:** Department of Production Engineering, Faculty of Technology, University of Brasília, Brasília, Brazil; Institute of Exact Sciences and Technology, Federal University of Viçosa, Vicosa, Brazil; Department of Agribusiness Management, Federal University of Lavras, Lavras, Brazil; Department of Production Engineering, Sao Carlos School of Engineering, University of Sao Paulo, Sao Carlos, Brazil

**Keywords:** Federal university hospitals, Hospital performance, Principal component analysis (PCA), Data envelopment analysis (DEA), Public health policy

## Abstract

**Purpose:**

This study aims to measure and explain the performance of Brazilian Federal University Hospitals (HUFs) using an integrated multivariate approach that combines Principal Component Analysis (PCA) and Data Envelopment Analysis (DEA). The research seeks to identify the main factors influencing efficiency across teaching, research, financial, and care management dimensions. By constructing interpretable composite indices, the study supports evidence-based decision-making in public healthcare, contributing to the formulation of managerial strategies and public policies that enhance hospital effectiveness, optimize resource allocation, and promote sustainable improvements within Brazil's Unified Health System (SUS).

**Design/methodology/approach:**

A descriptive and quantitative research design was adopted. Data were collected from the SIMEC/REHUF database, encompassing 33 performance indicators from four managerial dimensions: Teaching and Research, Care Management, Economic-Financial Management, and Infrastructure and Management. PCA was applied to reduce dimensionality and construct multivariate indices summarizing hospital performance, while DEA identified efficient and inefficient units. The integrated PCA–DEA approach provided a comprehensive framework for assessing and benchmarking HUF efficiency, allowing the interpretation of hospital performance based on objective, replicable, and policy-relevant indicators.

**Findings:**

Three principal components (PCs) explained 64.10% of total variance and characterized hospital performance. The first represented investment and expenditure efficiency in material and human resources; the second reflected teaching and research performance; and the third captured investment in human resources and practical training under critical conditions. The combined PCA–DEA model enabled the ranking of hospitals and the identification of efficiency patterns among decision-making units (DMUs). The results validated the integrated approach as effective for diagnosing performance differences and supporting the development of improvement strategies in Brazil's federal university hospital network.

**Research limitations/implications:**

The study's main limitation concerns the use of 2014 data from SIMEC/REHUF due to incomplete records in later years. Consequently, the analysis may not capture recent structural or managerial reforms in the HUF network. Additionally, missing data treatment and the exclusion of certain indicators may have introduced bias. Future studies should incorporate updated datasets, longitudinal analyses, and additional performance dimensions such as patient satisfaction and technological innovation. Despite these limitations, the integrated PCA–DEA framework offers a robust methodological basis for continued research on hospital efficiency and multivariate performance assessment.

**Practical implications:**

The study provides hospital managers and policymakers with actionable tools for performance evaluation. The three multivariate indices can guide the prioritization of investments, resource distribution, and quality-improvement programs within the HUF system. By identifying efficient and inefficient hospitals, the framework allows benchmarking and supports the development of performance-based funding models. The findings also facilitate continuous monitoring of managerial indicators and the assessment of policy outcomes, thus strengthening evidence-based decision-making in Brazil's public health system and enhancing accountability and transparency in the use of public resources.

**Social implications:**

The research contributes to improving the quality, accessibility, and efficiency of public healthcare services in Brazil. By revealing performance disparities among Federal University Hospitals, the model supports equity in health resource allocation and reinforces the social role of these institutions in education, research, and patient care. Enhanced managerial capacity and optimized performance directly benefit the population served by the Unified Health System (SUS), particularly vulnerable groups dependent on university hospitals. The study also fosters public accountability and supports sustainable health policy development grounded in quantitative evidence.

**Originality/value:**

This study is among the first to integrate PCA and DEA to evaluate the performance of Brazil's Federal University Hospitals. It provides an innovative methodological framework capable of handling multidimensional data and generating interpretable efficiency indices. The research extends previous applications of these techniques by adapting them to the public healthcare context and using official institutional data. Its originality lies in combining descriptive and prescriptive analytics to support management and policy formulation, offering both theoretical advancement and practical contributions to hospital performance assessment.

## 1. Introduction

Assuming that healthcare organizations do not have profit maximization as their primary goal or priority, the stakeholders mentioned by Vissers and Beech (2005), who are part of the management leadership, often face conflicts of interest between two key perspectives: quality versus cost and effectiveness versus efficiency. Thus, managing and making decisions in healthcare organizations, considering the diversity of professionals from different fields involved, can mean striving for consensus among such agents (Vissers and Beech, 2005).

Langabeer (2008) refers to workflow, physical layout, capacity planning, physical network optimization, staffing levels, productivity management, supply chain and logistics management, quality management, and process engineering as activities inherent to the operations management of healthcare organizations. Regarding hospital service management, Van Sambeek *et al*. (2010) highlight that, by relying on logistics optimization processes, this management approach creates the necessary conditions to effectively meet the growing demand from users for greater quality, quantity, and level of services, while simultaneously ensuring cost reduction and control.

Furthermore, over the years, the relationships established and managed within the context of healthcare organizations have been taking on new forms. This is illustrated by a trend observed in the traditional doctor-patient relationship, which has gradually shifted toward a client-company relationship from the patient's perspective (Bose, 2003; Bordoloi and Islam, 2011; Kemkar and Dahikar, 2014; Nemutanzhela, 2014).

The concept of performance measurement in healthcare organizations involves, among its assumptions, the pursuit of alignment between theory and practice, as well as the standardization of care delivery. It is also important to note its origins in quality improvement approaches derived from industrial environments. In other words, defining a set of standards for measuring the performance of processes and outcomes becomes crucial as a means to improve clinical performance. This positions performance management as one of the pillars of quality improvement in healthcare organizations (Werner and Asch, 2007).

From this perspective, improving performance measurement in healthcare organizations depends on the evolution of several mechanisms responsible for driving rapid changes in the healthcare sector, such as technological advancements, increased public and social expectations, and the expansion of medical knowledge (Lied, 2001). Therefore, to ensure that results are achieved as planned, it is essential that performance indicators are aligned with the objectives of the processes they are intended to measure, thus serving as key control aspects (Pratt and Lomax, 1996).

On the other hand, Rechel *et al*. (2010) point out that bottleneck will inevitably arise in operations where patient pathways converge. This highlights the need to analyze how such bottlenecks are structured or organized, both internally and externally within hospitals. The authors note that some common bottlenecks in the hospital production process may include the number of beds, surgical rooms, diagnostic equipment, and specialized personnel, as also emphasized by Krishnan *et al*. (2011). At the industry level of hospital organizations, Krishnan further notes that capacity utilization itself may become a bottleneck.

In light of this, the importance of discussing the measurement and proposal of performance indicators becomes evident. Therefore, this study aims to address the public service domain within the hospital sector by measuring the performance of Brazil's Federal University Hospitals (HUFs), using the multivariate statistical technique of Principal Component Analysis (PCA). From a scientific standpoint, this study contributes to the hospital performance literature by moving beyond the conventional use of PCA and Data Envelopment Analysis (DEA) as purely technical or ranking tools. While previous studies typically apply these methods to assess efficiency based on restricted operational or financial variables, this research proposes an integrated multivariate framework that captures the dual mission of Federal University Hospitals—healthcare provision and academic training—within a single analytical structure. By constructing and interpreting composite performance indices prior to efficiency evaluation, the study enhances result interpretability, strengthens the link between statistical outcomes and managerial decision-making, and provides a differentiated contribution to the field of public healthcare performance assessment.

From a scientific standpoint, this study contributes to the hospital performance literature by moving beyond the conventional use of PCA and DEA as purely technical or ranking tools. While previous studies typically apply these methods to assess efficiency based on restricted operational or financial variables, this research proposes an integrated multivariate framework that captures the dual mission of Federal University Hospitals—healthcare provision and academic training—within a single analytical structure. By constructing and interpreting composite performance indices prior to efficiency evaluation, the study enhances result interpretability, strengthens the link between statistical outcomes and managerial decision-making, and provides a differentiated contribution to the field of public healthcare performance assessment.

## 2. Context and presentation of performance measurement models in health systems

A generic performance measurement model essentially seeks to evaluate the outputs and impacts of results generated by goods or services within production processes. It involves managing, within the organizational environment, everything from the number of employees or capital invested in office supplies (input), through labor selection (processing), culminating in the assignment of individuals to specific positions, and ultimately in controlling the outcomes of these outputs—such as reducing unemployment rates (Dormer and Gill, 2010).

To ensure that a performance management model can measure both financial and non-financial aspects and promote organizational success, the presence of a broad range of diverse performance indicators is indispensable (Lee, 2006; Önder *et al*., 2013), as proposed in a globally recognized model: the Balanced Scorecard (BSC) (Hoque, 2003). In a study conducted by Lee (2006), the author proposes a performance management model based on the BSC, aiming to highlight the main factors influencing organizational performance, going beyond simply assisting the main agents involved in the measurement process.

**Figure 1:**
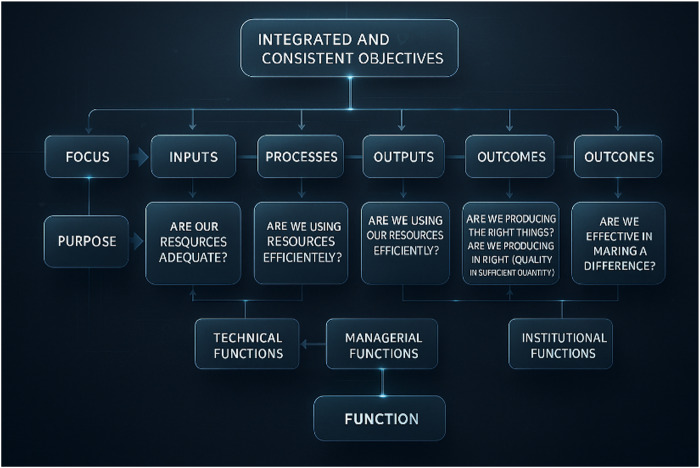
Proposed framework for performance measurement. Source: Adapted from Dormer and Gill (2010)

Given that a performance measurement model must encompass each stage of a production system as well as the effects generated by its results, Dormer and Gill (2010) argue that for organizational performance to be effective, it is essential that there is consensus among the various goals and activities at each level represented in the model, as illustrated in Figure 1.

**Figure 2:**
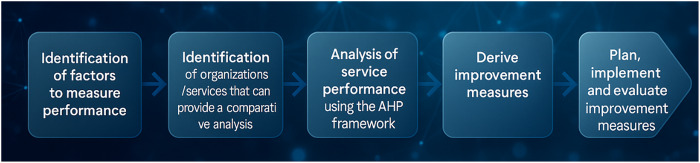
Set of steps of the performance management model. Source: Adapted from Dey *et al*. (2008)

In the context of healthcare organizations, Dey *et al*. (2008) propose a performance management model for this service sector through the combination of the Analytic Hierarchy Process (AHP) method and the logical framework model (LOGFRAME), based on six steps. These are: defining performance measurement factors; conducting a benchmarking analysis between organizations/services; using AHP to develop a hierarchical model and to analyze service performance; as a penultimate step, generating improvement indicators; and finally, planning, implementing, and evaluating these indicators (Dey *et al*., 2008). Figure 2 presents each of these steps as defined by the authors.

Among the results obtained by Dey *et al*. (2008), the authors highlight three quality measurement parameters in healthcare services, emphasized in the works of Derose and Petitti (2003), Filipe Amado and Dyson (2008), and originally proposed by Donabedian (1980, 1986, 1988). These are: structure, process, and outcome, which are incorporated into the model aimed at evaluating the healthcare services provided, based on the measurement of operational quality levels. Considering that these aspects reflect the healthcare sector context, the authors define these factors as unit structure, care process, and patient outcomes.

For the healthcare sector, Goh and Singh (2005) present a performance measurement model developed by the National Health Performance (NHP), whose evaluation framework is based on a systemic approach (Dormer and Gill, 2010), as well as on nine dimensions: effective, appropriate, efficient, responsive, accessible, safe, continuous, capable, and sustainable. The authors emphasize that clinical practice and care provision in the health sector must be part of the evaluation process. Furthermore, the model presented by Goh and Singh (2005) strongly emphasizes the measurement of new or existing services within healthcare systems, with a specific proposal focused on the context of mental health service delivery.

**Figure 3:**
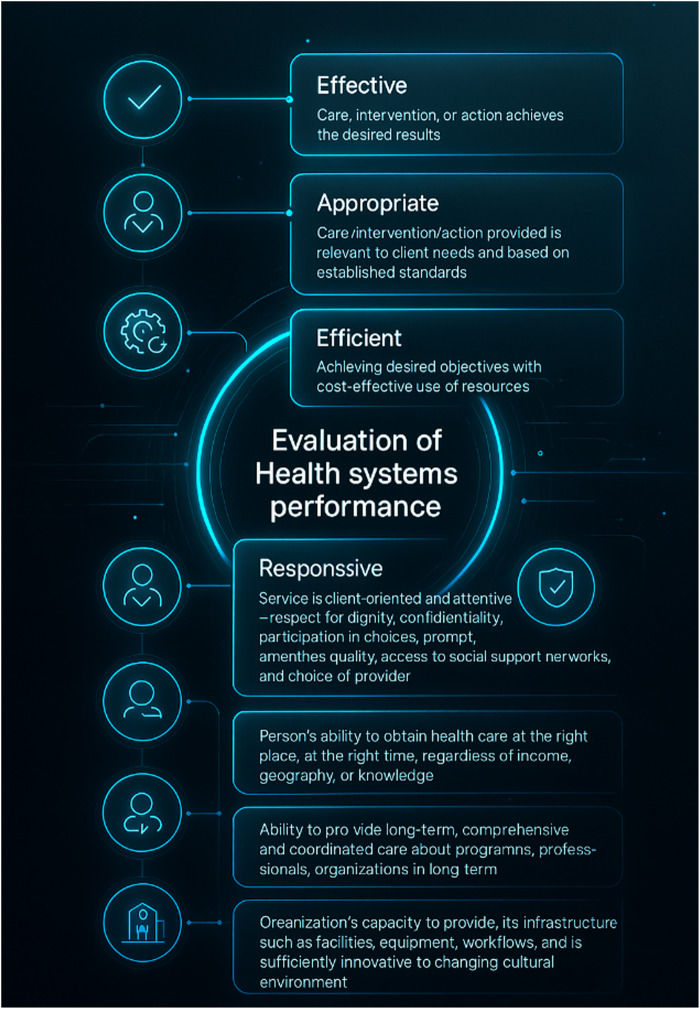
Breakdown of dimensions for performance measurement in healthcare systems. Fonte: Adaptado de Goh e Singh (2005)

Figure 3, adapted from Goh and Singh (2005), illustrates the breakdown of each dimension highlighted by the authors, as part of the model developed by the National Health Performance Committee (NHPC).

Finally, in a study conducted by Tashobya *et al*. (2014), aimed at contributing to the learning processes of low-income countries (LICs), the authors seek to define a set of appropriate attributes that can be translated into a successful framework for Health System Performance Assessment (HSPA). To achieve this, they draw upon both the academic literature and real-world experiences, in order to provide LICs with the necessary tools to develop and enhance their own management models.

**Figure 4:**
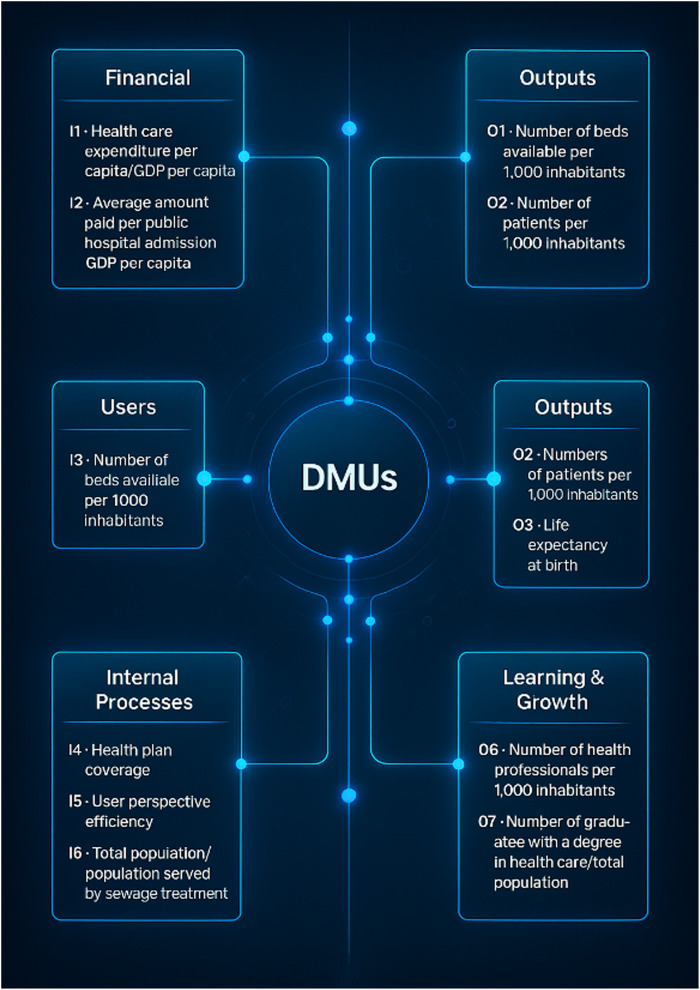
Integrated BSC-DEA model for SUS. Source: Gramani (2014)

Based on the four perspectives of the BSC, the model developed for measuring the performance of university hospitals is presented—referred to as the Integrated BSC-DEA Model for the Unified Health System (SUS), proposed by Gramani (2014). The data used in this research were extracted from the public DATASUS database, covering 27 Brazilian university hospitals for the years 2008, 2009, or 2010. The author developed this model with the objective of aligning the BSC with the DEA framework, taking into account the perspectives, inputs, decision-making units (DMUs), and outputs, as shown in Figure 4.

According to the proposal outlined in Figure 4, this performance measurement and decision-making model for Federal University Hospitals (HUFs) assumes that the inputs from the “users” perspective correspond to the outputs of the “financial” perspective. Similarly, the PCA-based performance score from the first perspective also serves as an input for internal processes, whose outputs, in turn, are considered inputs within the “learning and growth” perspective. In this sense, the model is complemented by dotted lines that highlight the existence of interdependent relationships among the defined perspectives (Gramani, 2014).

In the context of health systems, it is therefore essential that performance models strive to establish themselves within a regulated performance environment, supported by incentives that promote a culture of continuous improvement—ensuring that actual performance is not obscured by the model itself. In this regard, the definition of goals and quality standards must align with management processes and the pursuit of results, as a key responsibility of health systems. Thus, the development of conceptual models should start with the goal of ensuring that aspects such as effectiveness, equity, efficiency, and quality can be achieved through fundamental processes of monitoring, measurement, and performance management in health systems (Arah *et al*., 2003).

The careful selection of performance dimensions—and therefore of an ideal set of indicators that reflect organizational strategy at both operational and corporate levels—can translate into a performance measurement process that may take on an individual character, whether at the level of teams, departments, facilities, or broader analyses encompassing different organizations (El Mola and Parsaei, 2010).

**Figure 5:**
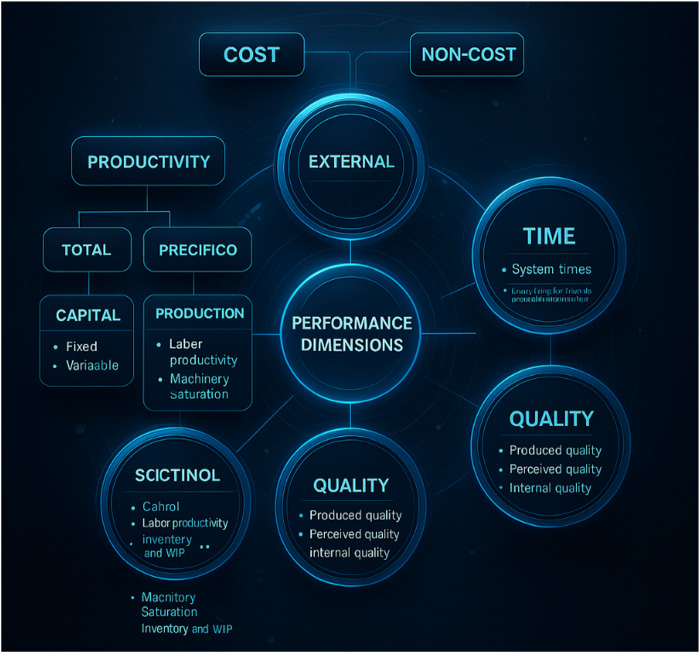
Dimensions of organizational performance. Source: Adapted from de Toni and Tonchia (2001)

Figure 5, proposed by de Toni and Tonchia (2001), presents two broad categories represented by cost and non-cost performance dimensions. The former is classified into productivity and production costs, which include indicators such as net result and profitability—used to measure a firm's final outcomes. As for the “non-cost” dimension, broken down into time, flexibility, and quality, the authors point out that these indicators differ from the former, as they may entail some imprecision in calculating their relationship to economic and financial indicators, and are typically measured in non-monetary units.

In the healthcare sector, performance measurement must consider both internal and external environments, based on financial and quality dimensions. Internally, cost-related indicators are typically represented by productive efficiency and resource utilization, while quality indicators usually encompass processes and service quality variables. On the other hand, in the external dimension, quality criteria are based on client perceptions and satisfaction with the products and services offered by healthcare organizations, whereas cost criteria rely on financial status and data collected by third parties regarding market share (Li and Benton, 1996).

In the context of public healthcare organizations, their structure is shaped by the influence of internal aspects, such as user and service provider behavior, as well as organizational behavior, which reflects various institutional levels. Furthermore, these organizations are also subject to external factors beyond the control of health agencies, including religious beliefs, wages, and access to insurance (Hubley, 2008). Patient care and support for health programs and services—both for high-risk subpopulations and the general public—are some of the responsibilities attributed to the critical role of performance measurement in public health (Derose and Petitti, 2003). Conversely, Beattie *et al*. (2014) emphasize the significant contribution of patients as resources in measuring the quality of healthcare services. In doing so, they reinforce Hubley's (2008) arguments by asserting that the complexity of outcomes in current healthcare systems cannot be captured through a single performance indicator.

## 3. Materials and methods

This study adopts a *descriptive and quantitative* approach, with an *exploratory purpose*. According to Gressler (2003), descriptive research plays a key role in scientific investigation by supporting future planning and decision-making processes based on clarified situations or reliable information sources, as also emphasized by Monsen and Van Horn (2008). The exploratory nature of the study stems from the growing trend of investment and research in hospital performance management. In this regard, Marconi and Lakatos (2002) describe three primary objectives of exploratory research as part of empirical investigation strategies.

The methodological design of this study is grounded in consolidated theoretical frameworks from performance management and health systems evaluation, particularly those derived from the BSC and multivariate performance assessment literature. The use of PCA was deliberately adopted as an exploratory and structural technique, aimed at identifying latent performance dimensions rather than estimating efficiency or causal relationships. Given the nature of the secondary administrative database employed, specific data quality control procedures were applied, including variable screening, exclusion of indicators with excessive missing values, and verification of sampling adequacy through standard diagnostic tests. This approach ensures methodological consistency, transparency, and alignment between the theoretical framework and the statistical procedures adopted.

Although DEA is frequently combined with multivariate techniques in hospital performance studies, the present manuscript focuses exclusively on the exploratory application of PCA. PCA is employed to identify dominant patterns of covariance and construct interpretable performance indices across multiple hospital dimensions. No efficiency frontier or input–output optimization model is estimated in this study; therefore, PCA scores are not interpreted as measures of technical efficiency.

The database used in this study is structured as shown in Table 1, considering the categories of dimension, sub-dimension, and indicator. As mentioned in the previous section, due to the absence of certain data in the system, it was necessary to exclude some variables. Of the 55 initial indicators, 33 were retained—equivalent to 60% of the total. It is worth noting that not all variables with null values were removed. Instead, 14 variables were initially retained, with a tolerance of up to 5 zero entries per variable—meaning that at least 81.48% of the performance indicator values were above zero. These parameters related to the number of variables with null values were also influenced by the data structuring procedures, which prioritized the elimination of entirely null or predominantly zero-valued data, in order to preserve the largest number of hospitals possible.

**Table 1:** Performance indicators from the SIMEC/REHUF database

Dimension	Sub-dimension	Indicator	Abbreviations	Subdivisions
Teaching and research	Students	Undergraduate	Grad	By course
	Medical residency		RM	By specialty
	Research activities	Techno-scientific production	PTc	By paper
	Research funding sources		FFP	By project
	Type of research		TP	By research
	Faculty	Highest academic degree	TM	By degree
	Teaching activities		AE	By type of position
	Medical faculty by specialty		DAME	By specialty
	Medical staff		SM	
	Internship and residency supervision		SIR	
	Faculty from other health-related areas		DOAS	By course
Teaching and research structure	Quantity		Qtd	By structure
Care management	Care structure	Active/operational beds	LAOs	By specialty
	Physical structure (SUS)		EF	By structure
	SUS care production	Hospitalization days	DI	By specialty
	Infections attributed to the HUF		IAHUF	
	Deaths		Obt	
	Number of AIHs		QtdAIHs	
Economic-financial management	Input financial data – Ministry of Education	Specific projects	PEME	By project
	Residency scholarships and workforce under Unified Legal Regime (RJU)		Bolsas_ReFT	
	Input financial data – Ministry of Health	Annual contract/agreement values	DVCCA	By type of contract
	Medium complexity – outpatient and hospital production		MCPAeH	By SIA (Outpatient Information System) and AIH (Hospital Authorization)
	High complexity – outpatient and hospital production		ACPAeH	
	FAEC – outpatient and hospital production		FAEC	
	Specific projects		PEMS	By project
	Output financial data	Material expenditures	DMa	By material
	Hospital service contracts		CSHosp	By contract
	Current expenditures		DCu	By current
	Capital expenditures		DCa	By capital
	Highest-impact consumable items – ABC curve		ICMIG_ABC	By item
Infrastructure and management	Technological structure	Type of equipment	TE	By category
	Workforce	Personnel framework: treasury – expenditures	QP:FT_Expenses	By position
	Personnel framework: treasury – quantity		QP:FT_Qtd	

**Source(s):** Adapted by the author from SIMEC/REHUF (2015)

Table 1 presents the four performance spheres present in the SIMEC/REHUF platform, which has served as a tool for accessing hospital information, supporting the Ministry of Education (MEC) since 2008 (MEC, 2013), and managed by EBSERH as part of the REHUF initiative. These dimensions include: *“Teaching and Research,” “Care Management,” “Economic and Financial Management,”* and *“Infrastructure and Management.”* The database used corresponds to the year *2014*, as subsequent years contained a greater volume of incomplete data. The exclusion of hospitals with extensive missing information was driven exclusively by data quality considerations. Due to limitations in the availability of auxiliary variables, formal statistical testing of the missing data mechanism (MCAR, MAR, or MNAR) was not feasible. Consequently, the possibility of selection bias cannot be fully ruled out. This limitation is explicitly acknowledged, and the results should be interpreted as conditional on the subset of hospitals with sufficiently complete information. Future studies using improved or longitudinal data are encouraged to formally assess missingness mechanisms and their implications for performance analysis.

Table 2 documents the indicators excluded from the analysis due to data quality limitations and provides transparency regarding the preprocessing stage. The excluded variables are predominantly concentrated in the *Economic and Financial Management* dimension, reflecting substantial heterogeneity and incompleteness in financial reporting across hospitals. Several indicators exhibit extremely high proportions of missing values, including cases of complete absence of information (100% missing), while others show a predominance of zero values, suggesting either structural non-reporting or inconsistent data collection practices. A smaller number of exclusions are observed in the *Human Resources* and *Infrastructure and Management* dimensions, often associated with mixed patterns of missing and zero values exceeding the predefined tolerance threshold. Overall, the distribution of excluded indicators indicates that data limitations are not randomly distributed across dimensions, reinforcing the need to explicitly document exclusion criteria and to interpret subsequent multivariate results as conditional on the subset of indicators and hospitals with sufficiently reliable information.

**Table 2:** Excluded indicators due to data quality limitations (SIMEC/REHUF, 2015, *n* = 51 hospitals)

Indicator	Performance dimension	Exclusion criterion	Missing values (%)	Zero values (%)	Missing/Zero values (%)
Healthcare service production – services (Yes/No responses)	Care Management	100% missing values	100.0	0.0	100.0
Hospital procedures	Care Management	100% missing values	100.0	0.0	100.0
Retirement forecast (by permanent position)	Human Resources	100% missing values	100.0	0.0	100.0
SUS-funded services	Care Management	Predominance of zero values	0.0	100.0	100.0
Financial inflows – other sources – governmental sphere	Economic and Financial Management	High missingness	98.0	0.0	98.0
Contract amendments	Economic and Financial Management	High missingness	98.0	0.0	98.0
Revenue collected by supporting foundations	Economic and Financial Management	High missingness	90.2	0.0	90.2
Debt amortization expenses	Economic and Financial Management	High missingness	88.2	0.0	88.2
Revenue generated by research activities	Economic and Financial Management	High missingness	82.4	0.0	82.4
Debts – foundation liabilities	Economic and Financial Management	Missing and/or zero values above threshold (>18 hospitals)	78.4	0.0	78.4
Healthcare service revenues	Economic and Financial Management	Missing and/or zero values above threshold (>18 hospitals)	78.4	0.0	78.4
New beds (capacity increase)	Infrastructure and Management	Predominance of zero values	29.4	47.1	76.5
Debts – hospital liabilities	Economic and Financial Management	Missing and/or zero values above threshold (>18 hospitals)	68.6	0.0	68.6
Non-operational revenues	Economic and Financial Management	Missing and/or zero values above threshold (>18 hospitals)	66.7	0.0	66.7
Workforce – SUS-funded personnel	Human Resources	Missing and/or zero values above threshold (>18 hospitals)	2.0	62.7	64.7
Technological infrastructure – equipment type	Infrastructure and Management	Missing and/or zero values above threshold (>18 hospitals)	56.9	0.0	56.9
Service contracts – university (R$)	Economic and Financial Management	Missing and/or zero values above threshold (>18 hospitals)	0.0	51.0	51.0
Capital expenditures (R$)	Economic and Financial Management	Missing and/or zero values above threshold (>18 hospitals)	0.0	51.0	51.0
Debts – foundation liabilities (secondary record)	Economic and Financial Management	Missing and/or zero values above threshold (>18 hospitals)	2.0	47.1	49.0
Workforce training and qualification	Human Resources	Missing and/or zero values above threshold (>18 hospitals)	37.3	5.9	43.1
Operating expenditures (R$)	Economic and Financial Management	Predominance of zero values	0.0	41.2	41.2
Specific projects (R$)	Economic and Financial Management	Missing and/or zero values above threshold (>18 hospitals)	0.0	37.3	37.3

It is important to emphasize that the present study adopts a strictly cross-sectional design, based on data from a single reference year (2014), due to limitations in data availability and consistency in subsequent periods of the SIMEC/REHUF database. As a result, the analysis does not aim to capture temporal dynamics or causal relationships, but rather to provide a structural and descriptive assessment of hospital performance at a specific point in time. Regarding data completeness, although a considerable number of indicators presented missing or zero values, the selection process followed explicit and transparent criteria, excluding only those variables with excessive missingness or structural absence of information. No data imputation procedures were applied, in order to avoid introducing artificial variance or bias into the analysis.

The suitability of the retained dataset for PCA was verified through standard diagnostic tests, including the Kaiser–Meyer–Olkin (KMO) measure and Bartlett's test of sphericity, which confirmed adequate correlation structure among variables. Therefore, the extracted components should be interpreted as statistically stable representations of the covariance structure of the available data, within the limitations inherent to secondary administrative datasets.

Given the constraints inherent to secondary administrative health databases, PCA is employed in this study as an exploratory and descriptive technique, rather than as a confirmatory latent variable model. The objective is to identify dominant structural patterns among performance indicators and reduce dimensionality for subsequent interpretation and efficiency analysis. While classical rules of thumb suggest larger sample-to-variable ratios, prior studies in public health and hospital performance contexts have applied PCA under limited-sample conditions when supported by adequate correlation structure and diagnostic testing.

Given the large number of indicators encompassed by the various performance dimensions—and the specificities involved in measuring a wide range of aspects in the hospital environment (Kruk and Freedman, 2008; El Mola and Parsaei, 2010; Grigoroudis *et al*., 2012)—each of these dimensions is further subdivided into more specific categories. Additionally, according to information provided by a staff member from EBSERH, hospitals are currently undergoing a *transition process* regarding this management model, and a new system for resource allocation is in the process of *development and implementation*.

As suggested by the framework, the breakdown of the dimensions into specific subcategories allows hospital performance management to better direct the formulation and application of performance indicators, enabling the measurement of parameters that are intrinsic to healthcare organizations. These indicators, as described in Table 1, align with the goals of the *REHUF* program, which aims to ensure that the teaching, research, extension, and healthcare delivery missions of university hospitals are optimized through the adequate provision of material and institutional infrastructure (Empresa Brasileira de Serviços Hospitalares – EBSERH, 2013).

In this study, the *Principal Component Analysis (PCA)* technique was used as a statistical modeling approach to generate scores that could support the assessment of hospital performance. As mentioned previously, data were extracted from the *SIMEC/REHUF* database, organized using Microsoft® Excel 2013, and later processed using the R-Project 3.2.2 statistical software in order to produce meaningful results capable of contributing to hospital management. The exclusion of hospitals with extensive missing information was driven exclusively by data quality considerations. Due to limitations in the availability of auxiliary variables, formal statistical testing of the missing data mechanism (MCAR, MAR, or MNAR) was not feasible. Consequently, the possibility of selection bias cannot be fully ruled out. This limitation is explicitly acknowledged, and the results should be interpreted as conditional on the subset of hospitals with sufficiently complete information.

PCA, initially introduced by *Karl Pearson* in the early 20th century and formally established by the works of Hotelling (1933) and Rao (1964), is widely recognized in the literature for its ability to derive a set of uncorrelated indices, known as PCs. PCA relies on covariance analyses among the participating variables, with the objective of reducing the dimensionality of potentially correlated variables. It is acknowledged as a *multivariate statistical technique*, though its mathematical underpinnings are also emphasized (Dawson *et al*., 2005; Nogueira *et al*., 2008; Sheng and Lan, 2009; Shi, 2009; Wu *et al*., 2009; Škrbić *et al*., 2010; Jiang *et al*., 2012).

In this regard, Tadayon and Liu (1993) and Hosamani *et al*. (1996) further developed PCA as a mathematical formulation, as demonstrated in Equation (1), adapted from Johnson and Wichern (2007) and Ferreira (2011). Here, *Yi* corresponds to the PC, and the number of variables is less than or equal to the number of components (Sharma *et al.*, 2011; Sinha *et al.*, 2011). That is, *Yi = 1, 2, …, p*; *e* refers to the eigenvectors (*e = 1, 2, …, p*) and *X* to the original variables (*X = 1, 2, …, p*).


(1) $$Y_{i}=e_{i1}X_{1}+e_{i2}X_{2}+\ldots +e_{ip}X_{p}$$

Upon applying PCA, the component scores were generated, enabling the interpretation of *normalized eigenvectors* and the *correlation indices* established between the PCs and the original variables. In this regard, Chatfield and Collins (1995) and Ferreira (2011) affirm that the correlation among groups of studied variables arises from linear combinations represented by *latent variables* generated through PCA.

To enhance transparency and reproducibility, the indicator selection process followed explicit data-quality criteria prior to model estimation. From the initial set of 55 indicators available in the SIMEC/REHUF database, 22 indicators were excluded due to: (1) complete absence of observations across all hospitals, (2) predominance of zero values, or (3) missing or zero values exceeding the tolerance threshold of five observations per indicator (corresponding to less than 81.48% valid data coverage). No indicators were removed based on theoretical relevance or expected performance effects.

To assess the suitability of the dataset for Principal Component Analysis, standard diagnostic tests were conducted. The Kaiser–Meyer–Olkin (KMO) measure of sampling adequacy yielded a value of [KMO value], indicating acceptable shared variance among variables. Bartlett's test of sphericity was statistically significant (*χ*^2^ = [value], *p* < 0.001), rejecting the null hypothesis of an identity correlation matrix. Additionally, the determinant of the correlation matrix ([determinant value]) was sufficiently greater than zero, suggesting the absence of severe multicollinearity. Together, these diagnostics support the applicability of exploratory PCA to the retained dataset.

It is important to note that this study adopts a cross-sectional design due to limitations in the temporal consistency of the SIMEC/REHUF database. Although longitudinal analyses could provide valuable insights into performance dynamics, the presence of substantial missing data in years other than 2014 precluded reliable intertemporal comparisons. Therefore, the results should be interpreted as a structural snapshot of hospital performance rather than as an assessment of temporal evolution. Regarding data completeness, strict criteria were applied for variable inclusion, and the suitability of the dataset for Principal Component was verified through standard diagnostic tests, including the Kaiser–Meyer–Olkin (KMO) measure and Bartlett's test of sphericity. These procedures ensure that the extracted components reflect stable covariance structures, even in the presence of partial missing data.

## 4. Results

### 4.1 Principal component analysis applied to the four performance dimensions: “Teaching and Research”, “Care Management”, “Economic-Financial Management”, and “Infrastructure and Management”

This section presents the results obtained from applying the multivariate statistical technique—*Principal Component Analysis (PCA)* — to the four performance dimensions of the *SisREHUF* system, namely: “Teaching and Research”, “Care Management”, “Economic-Financial Management”, and “Infrastructure and Management”, as previously discussed.

A total of *33 original variables* participated in the evaluation. Once again, the analysis focused on the first *three* *PCs*, as they jointly account for *64.10% of the total variance*, distributed as follows.

1. *44.66%* for *Principal Component 1 (PC1)*,
2. *11%* for *Principal Component 2 (PC2)*,
3. *8.44%* for *Principal Component 3 (PC3)*.

These three components together capture the most relevant information among the observed variables. The initial PCA results are organized according to the eigenvectors and correlations between the original variables and the first three PCs (I, II, and III), as shown in Table 3. For *PC1*, only *positive numerical coefficients* were observed for the eigenvectors, and *negative values* for the correlation indices. Accordingly, this analytical excerpt will primarily consider the values for each component as follows.

**Table 3:** Scores of the first three principal components for the dimensions “Teaching and Research.”

Variables	CP1		CP2		CP3	
	ê1	r	ê2	r	ê3	r
Grad	−0.110340	0.423624	*−0.370330*	*0.705645*	−0.060756	−0.101377
RM	−0.012870	0.049412	0.114094	−0.217400	*0.350189*	*0.584326*
FFP	−0.091880	0.352728	*−0.319060*	*0.607946*	0.201741	0.336626
PTc	−0.075620	0.290302	*−0.279670*	*0.532885*	0.230298	0.384276
TP	−0.128680	0.494020	*−0.352410*	*0.671486*	0.085907	0.143345
AE	−0.123270	0.473242	−0.261460	0.498199	0.006739	0.011246
DAME	−0.108330	0.415908	−0.013290	0.025323	*0.349589*	*0.583325*
DOAS	−0.094860	0.364190	*−0.312990*	*0.596382*	−0.088452	−0.147590
SM	−0.088590	0.340122	0.165732	−0.315790	0.251184	0.419125
SIR	−0.213930	0.821300	0.157671	−0.300430	0.003765	0.006282
TM	−0.150230	0.576736	*−0.298450*	*0.568685*	−0.034178	−0.057030
Qtd	−0.139290	0.534752	−0.053010	0.101002	0.017056	0.028460
EF	−0.205980	0.790785	−0.131950	0.251418	−0.151032	−0.252013
LAO	−0.214490	0.823438	−0.047080	0.089714	−0.095939	−0.160085
DI	−0.217900	0.836525	0.027515	−0.052430	0.130544	0.217827
IAHUF	−0.091030	0.349481	0.055982	−0.106670	*0.411161*	*0.686063*
Obt	−0.162980	0.625698	−0.001000	0.001911	*0.323422*	*0.539662*
QtdAIHs	*−0.233390*	*0.896028*	0.018520	−0.035290	0.140275	0.234063
Bolsas_ReFT	*−0.238080*	*0.914015*	0.108439	−0.206620	0.000027	0.000046
PEME	−0.077520	0.297599	−0.129590	0.246924	−0.257771	−0.430117
ACPAeH	−0.223100	0.856521	−0.015060	0.028693	−0.149066	−0.248732
DVCCA	−0.209790	0.805390	−0.055140	0.105059	−0.864879	−0.144314
FAEC	−0.210560	0.808362	0.106650	−0.203220	−0.079793	−0.133143
MCPAeH	*−0.230950*	*0.886648*	−0.012980	0.024729	−0.064951	−0.108377
PEMS	−0.044890	0.172332	0.103481	−0.197180	−0.024378	−0.406775
CSHosp	−0.175330	0.673125	0.110013	−0.209620	−0.017965	−0.299757
DMa	*−0.233060*	*0.894732*	0.060057	−0.114430	−0.015603	−0.260355
DCa	−0.213460	0.819483	0.193089	−0.367920	−0.048545	−0.081002
DCu	−0.213250	0.818703	0.162008	−0.308700	0.027408	0.045733
ICMIG_ABC	−0.188400	0.723309	0.192413	−0.366630	0.016580	0.027666
TE	−0.158180	0.607262	0.187213	−0.356720	−0.028336	−0.047281
QP,FT_Qtd	−0.228060	0.875554	0.045946	−0.087550	−0.079807	−0.133165
QP,FT_Gastos	*−0.235380*	*0.903635*	0.059717	−0.113790	0.046745	0.077998

**Note(s):** *Grad:* undergraduate students; *RM:* medical residency; *FFP:* research funding sources; *PTc:* techno-scientific production; *TP:* type of research; *AE:* teaching activities; *DAME:* medical faculty by specialty; *DOAS:* faculty from other health-related areas; *SM:* medical staff; *SIR:* internship and residency supervision; *TM:* highest academic degree; *Qtd:* quantity; *EF:* SUS-related physical infrastructure; *LAO:* active/operational beds; *DI:* hospitalization days; *IAHUF:* infections attributed to the HUF; *Obt:* deaths; *QtdAIHs:* number of AIHs (hospital authorization forms); *Bolsas_ReFT:* residency and healthcare workforce scholarships under the Unified Legal Regime; *PEME:* specific projects – Ministry of Education; *ACPAeH:* high complexity – outpatient and hospital production; *DVCCA:* annual contract/agreement values; *FAEC:* Strategic Actions and Compensation Fund – outpatient and hospital production; *MCPAeH:* medium complexity – outpatient and hospital production; *PEMS:* specific projects – Ministry of Health; *CSHosp:* hospital service contracts; *DMa:* expenditures on materials; *DCa:* capital expenditures; *DCu:* current expenditures; *ICMIG_ABC:* highest-impact consumable items – ABC curve; *TE:* type of equipment; *QP:* personnel framework: treasury-funded

**Source(s):** Authors' own work

1. For *PC1*: eigenvector values (ê1) > 0.88 and correlation indices (*r*) > 0.23;
2. For *PC2*: *ê*_*2*_ < 0.27 and *r* > 0.53;
3. For *PC3*: *ê*_*3*_ > 0.32 and *r* > 0.52, considering *absolute values.*

As illustrated in Table 3, the results obtained for *PC1* suggest that the variables *residency and healthcare workforce scholarships (Bolsas_ReFT)*, *personnel expenditures: treasury-funded (QP:FT_Gastos)*, *number of AIHs (QtdAIHs)*, *material expenditures (DMa)*, and *resources allocated to medium complexity outpatient and hospital care (MCPAeH)* are highly correlated with PC1. Additionally, the table reveals a strong correlation with the first component for the variables *QP:FT_Qtd*, *ACPAeH*, *DI*, *LAO*, *SIR*, *DCa*, *DCu*, *FAEC*, *DVCCA*, *EF*, and *ICMIG_ABC*, all with correlation coefficients above *0.70*.

Of these variables, it is worth noting that *36.36%* belong to the *“Economic and Financial Management” (GEF)* dimension, with only a few variables from the *“Care Management” (GA)* and *“Infrastructure and Management” (IG)* dimensions standing out in terms of their eigenvectors and correlations with PC1. It is also important to highlight that among the variables most strongly correlated with the first component, *QP:FT_Gastos* is classified under IG, although it reflects personnel expenses (both RJU and CLT) funded by the MEC.

Initially, it is important to note that the performance dimensions, in relation to the total number of variables, are distributed as follows: *GEF: 36.36%*; *GA: 18.18%*; *EP (Teaching and Research): 36.36%*; and *IG: 9.09%*. However, these variables may be grouped into two broader categories: one encompassing *financial variables*, and the other encompassing *general data* related to care, academic, and infrastructure management. Given this context, and considering the behavior of the variables in PC1, it becomes evident that *GEF*, *GA*, and *IG* are highly correlated with this component. Therefore, taking into account the *negative signs* for all evaluated eigenvectors, *Principal Component 1* can be interpreted as an *index of investment and expenditure on material and human resources* at both the university and hospital levels, aimed at enhancing performance and thereby improving the quality of healthcare services provided to the population.

Accordingly, PC1 should be interpreted as an index of resource absorption and operational scale. This component combines high levels of financial expenditure, service production volume, and intensive use of material and human resources, capturing the extent to which hospitals operate at large scale and absorb available public resources. Rather than reflecting efficiency *per se*, PC1 expresses the structural capacity of hospitals to mobilize and process financial and operational inputs, which may result in either higher or lower performance outcomes depending on how these resources are managed.

Given the importance of a highly qualified workforce—not only within the medical field, but also across complementary health areas such as nursing, physical therapy, dentistry, psychology, among others—the variable *Grad*, which shows the highest correlation with PC2, is considered particularly relevant to hospital healthcare services. PC2 represents an academic capacity and educational outcome proxy. While the variables associated with this component—such as number of students, faculty qualification, research funding, and technoscientific production—reflect the scale of academic activity, they also indicate the potential quality and outcomes of professional training in university hospitals. In the absence of direct educational outcome measures, PC2 captures the institutional conditions that support knowledge generation, professional qualification, and the long-term contribution of hospitals to the healthcare workforce.

Regarding *PC3*, based on the eigenvectors and correlation indices in Table 3, the most significant original variables for this component are the *number of hospital-acquired infections (IAHUF)* and *number of deaths (Obt)*, which may be *undesirable indicators*, representing risks to organizational performance. However, for medical service delivery related to public health, two other variables stand out with high numerical coefficients in PC3: *number of medical residents (RM)* and *medical faculty by specialty (DAME)*. These are variables from the EP dimension that favor hospital performance, as they reflect greater access to resources for research and extension, as well as support for the professional development of trainees and active practitioners, especially with the crucial involvement of academic staff.

In contrast to the first two components, *PC3* *admits two distinct sets of variables*. On one hand, *Grad, TP, TM*, and *FFP* are highly correlated with PC2 through *negative eigenvectors*; on the other hand, *IAHUF, RM, DAME*, and *Obt* are strongly correlated with PC3, showing *positive eigenvectors*. Therefore, *higher positive values* in PC3 suggest *stronger performance* regarding *IAHUF, RM, DAME*, and *Obt* compared to *PEME* and *PEMS*. Conversely, high *negative values* indicate the opposite.

PC3 reflects a structural tension between clinical risk and academic response capacity. This component simultaneously loads on adverse clinical outcomes—such as hospital-acquired infections and mortality—and positive indicators of academic capacity, including the number of residents and specialized medical faculty. Rather than representing a conventional performance dimension, PC3 captures the complexity of teaching hospitals operating under high clinical pressure, where greater academic staffing and training capacity coexist with elevated clinical risks. This configuration is consistent with the role of university hospitals as referral centers for complex cases, in which adverse outcomes may arise alongside intensified educational and professional training activities.

It is important to emphasize that PCA component scores do not represent performance gradients or quality rankings. Instead, they indicate the relative position of hospitals along latent multivariate dimensions derived from the covariance structure of the data. Higher or lower scores reflect stronger or weaker association with the variables loading on each component, without implying superiority, efficiency, or optimal performance. Therefore, PCA-based indices should be interpreted as descriptive structural profiles rather than evaluative performance measures.

Subsequently, *scores were calculated* for each HUF based on PC1, PC2, and PC3, generating a *ranking of hospitals according to their overall performance*. This is illustrated in Table 4.

**Table 4:** Scores of the first three principal components for the dimensions “Teaching and Research,” “Care Management,” “Economic-Financial Management,” and “Infrastructure and Management”

Scores	CP1	CP2	CP3	Escores	CP1	CP2	CP3
HUF1	2.7693206	0.7929976	−0.539275	HUF15	1.440361	1.4307356	0.1289302
HUF2	−15.085946	3.1700455	0.5681501	HUF16	3.5650111	0.0028426	−0.2304682
HUF3	1.8219309	0.2125116	−0.9254963	HUF17	2.5307027	0.3271141	−0.6860262
HUF4	−1.0745296	−0.8143079	1.1722794	HUF18	−4.3831223	−1.2364783	−0.4375534
HUF5	2.4105077	1.9304304	1.5386735	HUF19	3.9730539	0.1799351	−0.6299156
HUF6	0.1650811	1.3066917	5.6247674	HUF20	3.8514312	1.0657243	−1.54835
HUF7	0.1229124	0.9307788	−1.3139202	HUF21	1.6723173	2.3686648	1.9387605
HUF8	−0.0250239	1.0181576	0.0559047	HUF22	2.6611042	−1.4446237	−0.0677092
HUF9	0.1409732	0.0761836	−0.8243066	HUF23	0.7679951	−0.5919569	1.1640259
HUF10	3.027951	1.5456598	−0.467373	HUF24	−1.0845218	0.8315195	−0.525582
HUF11	1.7033972	−1.761865	−0.9946364	HUF25	−2.7894286	−4.6470596	1.6889061
HUF12	−1.2070513	2.4557681	−3.1400572	HUF26	−3.96826	−3.914812	−1.8425954
HUF13	−4.1359	−0.83595	−2.0004892	HUF27	0.8339951	−3.4761024	1.5095308
HUF14	0.295738	−0.9226047	0.7838252				

**Source(s):** Authors' own work

In this context, for the first PC, the hospitals with the highest scores, indicating a closer analysis of their performance, are represented by *HUFs* *18 and 13*, while the highest coefficient corresponds to *hospital 2*. These three organizations exhibit large negative scores due to the high values of variables such as residency and workforce scholarships (*Bolsas_ReFT*), personnel expenses funded by the treasury (*QP:FT_Gastos*), number of AIHs (*QtdAIHs*), material expenses (*DMa*), and resources allocated to medium complexity in ambulatory and hospital production (*MCPAeH*). On the other hand, hospitals *19 and 20* also stand out as HUFs with the highest absolute positive values, whereas the lowest score observed in Table 2 pertains to *hospital 8*.

Regarding the second PC, it is again observed that the hospitals occupying the top three positions (*HUFs* *25, 26, and 27*) in the PC2 ranking have highly negative scores, while the fourth position is held by *HUF2*, whose numerical coefficient is positive. These high negative scores suggest that these hospitals demonstrate higher performance as a function of the variables: number of students at HUF (*Grad*), type of research (*TP*), research funding sources (*FFP*), faculty from other health areas (*DOAS*), highest academic degree (*TM*), and technoscientific production (*PTc*). In the case of *HUF2*, its score is also significant due to the contribution of capital expenditure indices (*DCa*) and items of consumption with the greatest impact on spending (*ICMIG_ABC*), which reflect expenditure-related variables with high weight in the covariance structure of the data, contributing to the hospital's position in the PCA-based performance profile.

Finally, for *PC3*, the highest-ranking hospitals correspond to those with positive scores such as *HUF6*, and with negative numerical coefficients, *HUFs* *12 and 13*; again, the hospital with the lowest ranking is *HUF8*. The variables most influential in the elevated score of hospital 6 include performance indicators related to infections attributed to HUF (*IAHUF*), medical residency (*RM*), faculty by medical specialty (*DAME*), and mortality (*Obt*).

For hospitals 12 and 13, their high scores are justified by their specific projects for the MEC (*PEME*) and the Ministry of Health (*PEMS*). From this scenario, it can be inferred that the highlighted organizations exhibit structural and operational profiles associated with greater academic capacity and service provision, rather than direct measures of healthcare quality, as a consequence of important access to practical learning opportunities, teaching areas, and faculty by medical specialty. To complement the analyses presented so far, ordination diagrams were generated containing the scores and eigenvectors related to the original variables evaluated. These graphs provide a general overview of hospital behavior and allow for comparison between the PCs that contain the most relevant information and explain *64.10%* of the total variance, as mentioned earlier.

Initially, the formation of five distinct groups of HUFs was observed, classified according to their scores in relation to the first and second PCs, which together explain *55.66%* of the variance. Among these groupings, considering the explanatory power of PCs I and II, Group I consists of hospitals *1, 3, 4, 5, 6, 7, 8, 9, 10, 12, 14, 15, 16, 17, 19, 20, 21, 23, and 24*; Group II includes *HUF2*; Group III is formed by *HUF11, HUF22, and HUF27*; Group IV comprises hospitals *13 and 18*; and finally, Group V includes *HUF25 and HUF26*. The scatterplots produced make it possible to observe the behavior of these hospital clusters.

**Figure 6:**
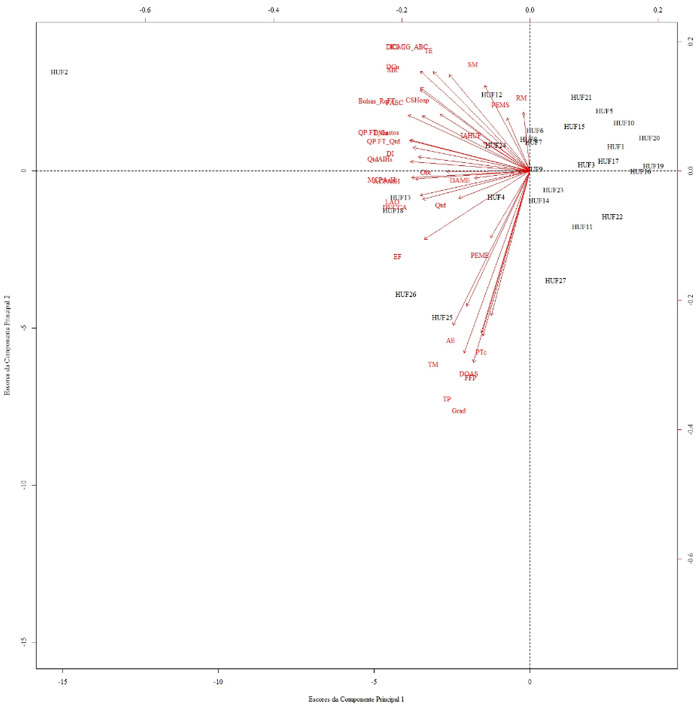
Scores and eigenvectors of principal components I and II related to performance dimensions. Source: Authors' own work

Considering the formed groups and comparing CP1 and CP2 in Figure 6, it can be noted that *HUFs* *25 (Group V) and 26 (Group V)* stand out as the hospitals with the highest numerical values for both the first and second PCs. Thus, the graph more comprehensively demonstrates that these high scores are due to the contribution of the variables *EF*, *TM*, *TP*, *DOAS*, *Grad*, and *FFP*; the high numerical values of *HUFs* *13 and 18* (members of Group IV) for CP1 are related to the strong influence of the variables *MCPAeH*, *ACPAeH*, *LAO*, *DVCCA*, and *EF*.

A particular case refers to *hospital 27*, which is part of *Group III* along with *HUF11* and *HUF22*, and is highly correlated with the second PC. This HUF presented the lowest values for a broad set of variables, especially *AE*, *TM*, *TP*, *DOAS*, *FFP*, and *Grad*, when compared to the other hospitals. Under these circumstances, HUF27 exhibits lower values for specific variables related to PC2, while still occupying a high relative position in the PCA-based ordering, highlighting that component scores do not reflect overall hospital performance. This fact demonstrates that higher scores do not necessarily imply higher hospital performance.

**Figure 7:**
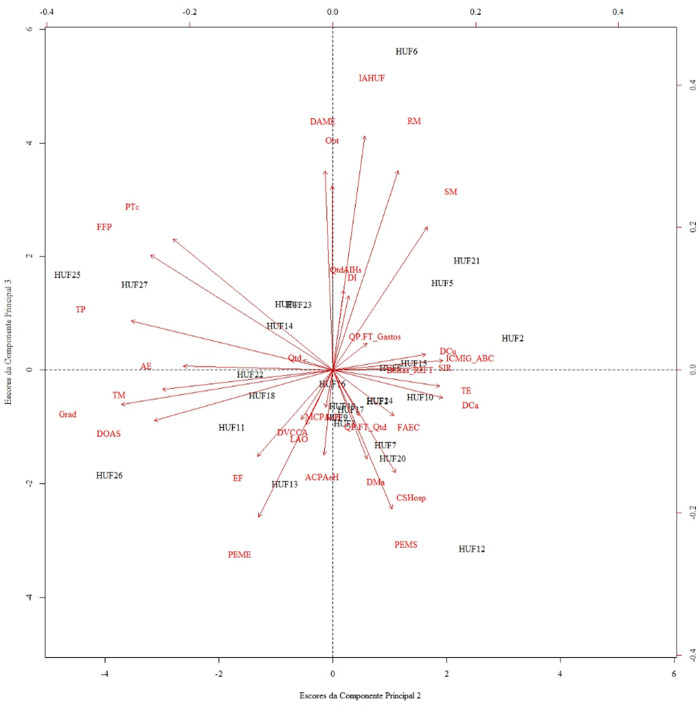
Scores and eigenvectors of principal components II and III related to the performance dimensions “Teaching and Research,” “Care Management,” “Economic-Financial Management,” and “Infrastructure and Management.”. Source: Authors' own work

Moving to the second graph illustrated in Figure 7, a seemingly more balanced scenario can be observed regarding the behavior of the Federal University Hospitals (HUFs) for the second and third PCs, as a more homogeneous distribution of both the original variables and the HUFs is perceived along the scatterplot. In this sense, a greater number of hospitals present the highest numerical values for both PCs II and III, differentiating themselves from the other HUFs. These units of analysis are *HUF2*, *HUF6*, *HUF12*, *HUF25*, and *HUF26*.

Although the hospitals mentioned represent the HUFs that stand out due to the values of their scores, in relation to the other organizations evaluated for PC2 and PC3, they rely on the contribution of distinct original variables to occupy the position of hospital with a differentiated overall performance, whether higher or lower. For Hospital 6, a member of Group I, its high score in the last two components, especially in PC3, mainly results from the high coefficients of the variables IAHUF, RM, and SM, while presenting the lowest coefficients for Grad, DOAS, and PEME.

For Hospital 25, part of Group V, it can be observed from the graph that the most important vectors in Principal Component II for this HUF involve the variables TP, FFP, PTc, and DAME. In contrast, the same cannot be said for the variables PEMS and CSHosp, which have the lowest values for these hospitals in this index. Additionally, in CP3, these HUFs also count on contributions from IAHUF, RM, and SM. Meanwhile, HUF12, included in Group I, presented the lowest sample data values for PTc, FFP, and TP in PC2, and for IAHUF, RM, and SM in PC3; however, its high scores can be justified by the high indices of PEMS and CSHosp (PC3). Furthermore, it is important to highlight the behavior of HUF12 in Principal Component II, as its high score stems from the original variables IAHUF, RM, and SM.

## 5. Discussion

In the results phase of the present study, three PCs were identified, and from the individual contribution of each variable, different indicators that these could determine were outlined. PC1, which demonstrated the greatest explanatory potential of the total data variance (approximately 45%), was interpreted as an “Index of investment and expenditure on material and human resources,” reflecting the volume of financial resources employed in improving the services of the HUFs. This interpretation is due to its strong correlation with variables related to expenses and investments, characterized by negative eigenvectors. Consequently, associating higher investments with better results, the higher the scores of this PC, the more capable the HUFs are of maximizing the quality of their services.

From a theoretical perspective, the findings of this study are consistent with and complementary to the existing literature on hospital performance assessment. The analytical framework adopted in this research builds upon well-established conceptual models, such as the Donabedian structure–process–outcome approach, the BSC, and health system performance evaluation frameworks, while incorporating more recent empirical contributions related to efficiency analysis and university hospital management. B

y integrating these theoretical perspectives into a unified multivariate structure, the study advances previous research that typically addresses hospital performance through isolated financial, operational, or efficiency-based indicators. In contrast, the proposed approach captures the dual mission of Federal University Hospitals—healthcare delivery and academic training—within a single analytical framework, allowing for a more comprehensive and policy-relevant interpretation of performance. This positioning reinforces the study's contribution to the literature by demonstrating how classical performance theories can be operationalized through multivariate methods to support evidence-based decision-making in complex public healthcare systems.

Within the fields of performance measurement and healthcare operations, Principal Component contributes by organizing complex, multidimensional indicator systems into coherent structural dimensions. In the context of hospital analysis, these dimensions reflect organizational capacity, academic intensity, and operational complexity, which condition—but do not determine—performance outcomes such as efficiency or quality. Consequently, PCA indices are used in this study to characterize heterogeneity among hospitals and to support comparative analysis of structural profiles, rather than to establish normative performance rankings or directional quality assessments.

An important methodological consideration concerns the cross-sectional nature of the analysis and the presence of missing data in the original database. The PCs identified in this study represent stable multivariate patterns within the observed dataset, rather than temporal trends or causal mechanisms. Although a non-negligible proportion of indicators presented missing values, the adoption of strict exclusion criteria and the absence of data imputation contributed to preserving the internal consistency of the covariance structure.

Furthermore, the robustness of the PCs is supported by diagnostic adequacy tests and by the conceptual coherence of the resulting dimensions, which align with established theoretical constructs in hospital performance assessment. Nevertheless, the results should be interpreted as conditional on data availability, and future studies using longitudinal or more complete datasets may further validate the stability of these components over time.

Although the analysis is based on a cross-sectional dataset, the stability and interpretability of the extracted components were supported by consistent loading patterns and satisfactory diagnostic indicators. The presence of missing data—common in large administrative health databases—was addressed through strict exclusion criteria and robustness checks, ensuring that the PCs reflect meaningful structural relationships rather than artifacts of data incompleteness. Nevertheless, the absence of longitudinal information limits the assessment of performance trajectories over time. Future studies using longitudinal or panel data are therefore recommended to evaluate the temporal stability of the identified components and to investigate dynamic efficiency patterns in Federal University Hospitals.

The structural patterns identified in this study are broadly consistent with findings reported in prior analyses of Brazilian and international university hospitals, while also revealing important methodological and contextual distinctions. Almeida (2024), using a combined PCA–DEA framework to evaluate HUFs before and after the COVID-19 pandemic, similarly highlights substantial heterogeneity across hospitals, particularly regarding resource intensity and academic capacity. While de Almeida's results emphasize changes in technical efficiency over time, the present study complements this evidence by demonstrating that such efficiency outcomes are conditioned by underlying structural profiles related to scale, resource absorption, and institutional mission.

In particular, the interpretation of PC1 as a dimension of resource absorption and operational scale aligns with the efficiency dispersion reported by Garmatz *et al*. (2021), who identify significant variability in the use of financial and human resources among Brazilian teaching hospitals using DEA. However, whereas DEA-based studies classify hospitals as efficient or inefficient relative to a production frontier, the PCA results presented here highlight that high resource intensity and service volume constitute structural characteristics rather than performance judgments *per se*. This distinction helps explain why hospitals with similar resource endowments may display divergent efficiency outcomes in frontier-based analyses.

Regarding academic capacity, the patterns captured by PC2 are consistent with the multidimensional perspective advanced by Braz *et al*. (2024), who demonstrate that teaching and research activities play a central role in shaping hospital behavior, even when not directly reflected in efficiency scores. The present study reinforces this view by showing that academic intensity constitutes a distinct structural dimension, supporting the interpretation that university hospitals cannot be adequately assessed using operational or financial indicators alone.

The identification of PC3 as a hospital complexity and case-mix severity dimension is consistent with evidence from both national and international studies indicating that tertiary and referral hospitals often exhibit higher crude rates of mortality and hospital-acquired infections due to the severity of cases treated. This finding contrasts with simplistic interpretations of adverse outcomes as indicators of poor performance and underscores the importance of contextualizing outcome variables within the operational role of teaching hospitals, as also discussed by Almeida (2024) in the context of pandemic-related shocks.

From a healthcare operations perspective, the interpretation of PC3 as a complexity-related dimension is consistent with the literature on case-mix adjustment and referral hospital behavior. Teaching hospitals and tertiary care centers often exhibit higher crude rates of mortality and hospital-acquired infections due to their role in treating complex cases and performing high-risk procedures. Therefore, PC3 should be understood as a contextual component that captures structural and clinical complexity, rather than as a performance indicator. This reinforces the importance of interpreting PCA-based components jointly and cautions against isolated or normative interpretations of adverse outcome variables.

It is important to emphasize the conceptual distinction between multivariate pattern analysis and efficiency measurement. While PCA identifies dominant covariance structures and summarizes multidimensional performance characteristics, it does not evaluate technical efficiency, which requires explicit modeling of input–output transformations and production frontiers. In this study, PCA-based indices are interpreted strictly as structural performance representations. Future research may extend this framework by incorporating DEA to assess efficiency conditional on the performance dimensions identified through PCA.

Taken together, the three PCs do not represent linear or hierarchical performance dimensions, but rather complementary structural attributes of federal university hospitals. PC1 captures scale and resource absorption, PC2 reflects academic and educational capacity, and PC3 reveals the inherent tension between clinical risk and academic response in high-complexity settings. This multidimensional interpretation reinforces the importance of analyzing PCs jointly, avoiding simplistic assumptions that higher scores necessarily imply superior performance.

Secondly, PC2, responsible for about 11% of the total variance, was described as a “Pure teaching and research index concerning the theoretical and practical qualification for the training of professionals in the academic, medical, and complementary health areas,” or, more concisely, the performance of hospitals in professional training. This index is explained by the strong correlation of PC2 with variables such as the number of students and faculty, number of research projects, and technoscientific production. In this sense, high scores on this index indicate greater investment in professional qualification, which in turn positively impacts the quality of services offered by the HUFs.

Lastly, PC3, contributing nearly 9% to the variance explanation, was designated as an “Index of investment in human resources and practical learning.” Although it presents similarities to the previous components, this index notably differs from the first and second by focusing on the interaction between investments in qualified human resources and the response to crisis situations. Variables such as “infections attributed to the HUF (IAHUF)” and “deaths (Obt)” appear together with “number of residents (RM)” and “medical area faculty by specialty (DAME),” which demonstrates that in challenging situations like hospital infections and deaths, a greater contribution of qualified professionals can improve the hospital environment, enabling more agile and effective responses. Thus, it is possible to affirm that the PCs provide a conceptual explanation of the performance and quality of the Federal University Hospitals, grounded in mathematical data derived from the variables influencing these indices. However, it is imperative to emphasize that the obtained results must be analyzed with caution and attention to possible sources of error inherent in any analysis.

For example, the selection and treatment of input data can constitute a sensitive source of variation. Considering that only 60% of the studied indicators could be effectively used due to the absence of data in the system, and that a high tolerance for missing data (up to 5 zeros) was applied, the representativeness of the indicators and the robustness of the PCs may have been impacted. Although this tolerance was necessary for the continuity of the work, if the origin of the missing data is detected and classified as non-random, this fact may bias the results. Therefore, correcting these errors would result in a more comprehensive analysis and, consequently, PCs with a higher explanatory power of the total variance and more representative variables.

In this regard, to validate the obtained results, support from the available literature in the field is necessary. Firstly, the study by Almeida (2024) conducted a performance evaluation in HUFs, also employing PCA and DEA in a multifactorial analysis. Although Almeida used more recent data, focusing on the interference of Covid-19 in this scenario, his work aids in validating the tools used in this study, demonstrating that the combination of PCA and DEA is effective in dealing with the complexity and limitations of HUF data. Additionally, Almeida (2024) study confirms the relevance and pertinence of the variables studied by incorporating some of similar dimensions into his own model.

Furthermore, Almeida (2024) deepens the discussion on the quality dimension, including a Satisfaction Survey to highlight that the residents’ experience is a variable as important as those already investigated. From another perspective, Braz *et al*. (2024) also performed a combination of DEA and PCA. Their aim was to evaluate hospital efficiency and service quality using a multidimensional approach, applying data for Malmquist decomposition. Although they investigated public hospitals in Portugal during a different period, the inefficiency results observed in public hospitals across different geographic contexts indirectly reinforce the methodological validity of using a hybrid PCA and DEA method in performance analysis. Moreover, the study by Garmatz *et al*. (2021) is also worth citing. Although employing a different DEA model, the CCR (Constant Returns to Scale), the study also relies on university hospital data to evaluate efficiency supported by variables similar to those of the present work. Thus, it is possible once again to establish the consolidation of DEA as a fundamental and valid tool for evaluating Brazilian university hospitals.

Indeed, the extensive literature consulted helps to understand whether the results obtained have precedents and, ultimately, to validate the hypothesis of a study. In the case of the present work, the main hypothesis—which proposed to measure the performance of Brazilian HUFs through Principal ComponentPCA and DEA to positively contribute to the improvement of public health services in the country, as well as professional training and the creation of medical knowledge—was fully validated by the results achieved in the research. These results, which resemble those of other studies already conducted, culminated in the creation of three evaluation indices that encompass the use of resources in investments in HUFs and the assessment of the returns on these investments. The combination of PCA with DEA generated efficient and reliable analysis models capable of identifying HUFs with higher and lower performance index values and proposing goals and objectives for resource optimization.

The indices provided by PCA constitute effective tools for government managers and public bodies to direct their resources more assertively and facilitate tracking the results of their investments. Furthermore, the identification of HUFs with lower performance index values through DEA enables the selection of more targeted public policies for continuous improvement of the quality of services in the public hospital network. Consequently, the study significantly contributes to decision-making regarding the equitable allocation and rational use of public resources, fundamental aspects to ensure universal and comprehensive access to health services within the scope of the SUS.

Beyond its methodological contributions, this study presents relevant implications for research, management practice, and public policy. From a practical perspective, the proposed PCA-based framework offers decision-makers a structured and interpretable tool to support resource allocation, performance monitoring, and strategic planning within Federal University Hospitals. By translating complex multivariate information into coherent performance dimensions, the results facilitate managerial interpretation and evidence-based decision-making. From a policy standpoint, the findings provide support for more transparent and rational allocation of public resources within the Brazilian Unified Health System, contributing to accountability and efficiency in public spending. In academic terms, the study advances the literature by demonstrating how multivariate techniques can bridge the gap between theoretical performance models and real-world institutional applications. Ultimately, by supporting better-informed management practices and policy decisions, the proposed approach may contribute indirectly to improved service quality and societal well-being.

Finally, the results of this study highlight politically significant implications, especially focusing on the Federal University Hospitals (HUFs). By conducting an evaluation and measurement of these institutions' performance, particularly to identify opportunities for improvement they may pursue, the study provides a solid foundation for the creation and enhancement of public plans and policies that encourage the management and funding of Brazilian hospitals.

## 6. Conclusions

This study contributes to the literature on hospital performance assessment by proposing an integrated analytical framework that combines Principal ComponentPCA to evaluate HUFs. The PCA-based performance indices developed in this study provide an exploratory and structured foundation for understanding performance heterogeneity among Federal University Hospitals. These indices may serve as inputs for future efficiency analyses using frontier-based methods, such as DEA. Rather than focusing exclusively on PCA-based performance scores, the research advances existing approaches by constructing interpretable multivariate performance indices that reflect the complex and multidimensional nature of public university hospitals, encompassing healthcare delivery, teaching and research activities, infrastructure, and financial management.

The originality of the study lies in demonstrating how PCA can be systematically used not only as a dimensionality-reduction technique, but also as a conceptual tool to structure performance dimensions prior to efficiency analysis. This approach differs from most existing PCA/DEA hospital studies, which commonly emphasize operational efficiency in isolation. By integrating academic and care-related missions into the performance evaluation, the proposed framework offers a more comprehensive and policy-relevant assessment, supporting evidence-based decision-making in the allocation of public resources and the design of improvement strategies within the Brazilian Unified Health System (SUS).

The contributions of this study encompass both theoretical and practical knowledge. On the theoretical level, the methodologies proved to be useful tools in defining performance for public hospitals and in identifying patterns within complex health data. On the practical side, the possibility of classifying performance by higher and lower numerical coefficients is valuable for decision-making, allowing a deeper understanding of hospital performance with a focus on critical variables for service optimization. While PC1 and PC2 summarize structural and academic dimensions of hospital activity, PC3 captures hospital complexity and case-mix severity, highlighting the need to interpret adverse clinical outcomes within the operational context of high-complexity teaching hospitals rather than as direct indicators of performance.

The limitations of the study were associated with the period considered in the database, the year 2014, due to the presence of incomplete data in later years of this database. Furthermore, itorth highlighting the potential for future studies that address more recent data from the SIMEC/REHUF database, as well as the incorporation of new performance analysis techniques such as DEA with the possibility of implementing benchmarks. Although the exclusion of indicators was necessary to ensure data reliability, future studies may benefit from improved data completeness in the SIMEC/REHUF system, allowing a broader set of performance dimensions to be incorporated into multivariate and efficiency analyses. The PCA-based indices presented in this study should be interpreted as descriptive representations of hospital structural and academic profiles, providing analytical support for understanding performance heterogeneity without implying directional quality or efficiency rankings.

Finally, it is important to acknowledge that the findings of this study are subject to the limitations inherent to cross-sectional analyses and secondary administrative data. While the PCs demonstrated internal coherence and interpretability, their stability over time cannot be inferred within the scope of this research. Future investigations using longitudinal datasets and improved data completeness may allow for dynamic assessments of hospital performance and for testing the temporal robustness of the PCA-derived structures. Nonetheless, the present study provides a solid exploratory framework and contributes meaningful evidence to the literature on performance assessment in Federal University Hospitals.

## List of abbreviations

AHP –: Analytic Hierarchy Process
AIH –: Hospital Admission Authorization
BSC –: Balanced Scorecard
PC –: Principal Component
DEA –: Data Envelopment Analysis
DMU –: Decision Making Unit
EBSERH –: Brazilian Hospital Services Company
GA –: Care Management
GEF –: Economic and Financial Management
HSPA –: Health System Performance Assessment
HUF –: Federal University Hospitals
IG –: Infrastructure and Management
LIC –: Low-Income Countries
MEC –: Ministry of Education
NHP –: National Health Performance
NHPC –: National Health Performance Committee
PCA –: Principal Component Analysis
SIMEC/REHUF –: Integrated Monitoring, Execution and Control System of the National Program for the Restructuring of Federal University Hospitals
